# English County Lunatic Asylums

**Published:** 1850-10-01

**Authors:** 


### ENGLISH COUNTY LUNATIC ASYLUMS.

It is the earnest desire of the Editor of this Journal to make it a medium of communication for all persons interested in medical psychology, or engaged in the care and treatment of the insane, and at the same time to afford such information as may conduce to a correct estimate, both in England and abroad, of our practice and progress in this department of medical science. For believing, as we do, that insanity is a disease of somatic origin, and, in its early stage, generally amenable to medical treatment, we consider it as direct and natural a subject for medical investigation as a fever or a pleurisy. We regard lunatic asylums as hospitals for the cure of the insane; and although, unfortunately, the obscurity of the disorder, its obstinacy, and the circumstances which prevent its early recognition and the adoption of curative treatment, too frequently convert them into mere houses of detention, still we must not lose sight of the fact that, in their primary intention, they are hospitals devoted to the cure of one of the most dreadful maladies to which mankind is liable.

The medical report of a lunatic asylum should, therefore, resemble a hospital case-book; it should contain a careful summary of the symptoms presented by the patients on admission, together with the history of the case; it should indicate the mode of treatment adopted, with the results of such treatment, marking the success or failure of the curative means employed; it should also contain a record of the morbid appearances observed in post-mortem examinations, and such general remarks as might serve to elucidate the subject. We do not mean to say that the report should contain the full particulars of each individual case, but that it should offer such a general summary of the particulars we have indicated as might serve for increase of knowledge and improvement of practice.

That the reports before us do not meet our demands—that the majority of them fall short in one or more points of the particulars we have indicated—is, we are fully aware, to be ascribed, not to any want of willingness, or zeal, or intelligence, on the part of the different medical officers who have assisted in preparing them, but to their servile, uninfluential position, the amount of service required of them, and the inadequate assistance afforded them. We also doubt whether many governing bodies of our public asylums could be found who would sanction the publication of such reports as we think needed; for we fear that the cost of printing would influence them more than any prospective benefit.

It was a common thing formerly to say—"They manage these things better in France," but the present Number of the Journal shows the error of that saying, as applied to the management of public asylums; but so far as reports are concerned we may with justice assert that—"they manage these things better in the United States." We have had occasion from time to time to notice some of the Transatlantic Reports, and we unwillingly acknowledge that, in many essential points, they are greatly superior to most of the pamphlets under notice. An improvement which would add considerably to the practical value and utility of our reports, would be the adoption of one uniform plan of matter and arrangement: at present, no two reports are drawn up in the same manner, and in many the arrangement is very inconvenient and complicated. We would also suggest the mutual interchange of reports between the resident medical officers of the various asylums.

We have before us Reports of the Yorkshire (N. and E. Ridings), York City, Suffolk, Lancaster Duchy, Liverpool Borough, Gloucestershire, Cheshire, North Wales, Devonshire, Somersetshire, Cornwall, Northamptonshire, Leicestershire, Nottinghamshire, Staffordshire, Dorsetshire, Bedfordshire, Oxfordshire, Lincolnshire, and Surrey Asylums; for which we are indebted to the kindness of the resident medical officers of these establishments, and to whom our best thanks and acknowledgments are due.

Of the foregoing twenty institutions, eight are pauper and twelve general asylums. On the 1st day of January, in the present year, they contained 5281 patients—2304 males, 2538 females, and 439 whose sex is not distinguished. These figures show an excess amounting to nearly ten per cent. of females over males. The number of patients treated in these establishments during the year 1849, amounted to 6781. Of these, 685 were discharged recovered, 135 relieved, and 512 died. These numbers give an average of 10·1 per cent. of cures, and 7·5 of deaths. The average number of recoveries was greater in the general than in the pauper asylums. Taking the Cornwall, Lancashire, Oxfordshire, Somersetshire, Suffolk, and Surrey, to represent the pauper—and the York City, Devonshire, Gloucestershire, Lincolnshire, Nottinghamshire, Staffordshire, to represent the general asylums, we find 10·8 per cent. of cures in the latter, to 8·8 per cent. of cures in the former. The number of deaths also differ, being 8·6 per cent. for the pauper—5·6 for the general asylums. We subjoin a table giving the total number of patients treated during the year, with the per-centage of recoveries and deaths.

| | No. of Patients. | Cures per cent. | Deaths per cent. |
|---|---|---|---|
| Yorkshire | 204 | 15·2 | 5·4 |
| York City | 181 | 9·3 | 6· |
| Suffolk | 342 | 12·8 | 8·7 |
| Lancaster | 980 | 6·4 | 9·1 |
| Liverpool | 106 | 18· | 10·3 |
| Gloucester | 391 | 11·5 | 5·8 |
| Cheshire | 245 | 9· | 4‘ |
| North Wales | 142 | 12·6 | 7· |
| Devonshire | 462 | 10. | 6·5 |
| Somersetshire | 284 | 9· | 6· |
| Cornwall | 258 | 8·5 | 11·6 |
| Northamptonshire | 370 | 10·2 | 6· |
| Leicestershire | 232 | 16· | 6· |
| Nottinghamshire | 344 | 11· | 6· |
| Staffordshire | 301 | 11· | 3·6 |
| Dorsetshire | 191 | 8·9 | 9·4 |
| Bedfordshire | 332 | 8·4 | 12· |
| Oxfordshire | 382 | 12·8 | 10·2 |
| Lincolnshire | 193 | 11·9 | 4·6 |
| Surrey | 841 | 8· | 7·2 |

We observe that in some reports the number of recoveries is calculated on the number of admissions during the year; in some, on the average daily number of residents; in others, on the total number treated during the year; we have preferred the latter mode, as being equally just to the different establishments, and affording a more correct criterion of the real curability of the disease. Suppose an asylum contains, on January 1st, 250 patients, and admits 50 patients during the year, the total number treated during the year will be 300; if of these 45 recover and 15 die, the recoveries and deaths will be respectively 15 and 5 per cent., and the average chance of recovery 1 to 6·6.

The expense for maintenance and treatment of patients varies considerably in different asylums, ranging from about 6*s.* 6*d.* to 11*s.* per patient per week, as is seen in the following examples:—

| | Cost per head per Week. s. | d. |
|---|---|---|
| Cornwall ... ... ... ... ... ... ... | 6 | 7½ |
| Yorkshire ... ... ... ... ... ... ... | 7 | 1¼ |
| Bedfordshire... ... ... ... ... ... | 7 | 6 |
| Oxfordshire ... ... ... ... ... ... ... | 9 | 5¼ |
| Lincolnshire... ... ... ... ... ... ... | 10 | 8½ |

This shows a very considerable difference, which may be partly owing to some variety in the method of keeping the accounts.

It is gratifying to observe the steady diminution in the contract price for all sorts of provisions and necessaries, during the successive quarters of the year 1849.

We have purposely excluded Hanwell Asylum from the preceding calculations, for in its present crowded state it is devoted to the care, rather than the cure of, the insane. Several of the Reports before us contain a great deal of valuable information. When so much industry and talent are exhibited, it is almost invidious to paticularize; but the Lincoln, Hanwell, and Dorset appear to us to merit especial notice, the Lincoln being, in fact, a very valuable treatise on the management of lunatic asylums. The Surrey and Littlemore (Oxford) contain useful statistical calculations.

We have received also the following reports of Scotch lunatic asylums —Aberdeen, Edinburgh, Dundee, Murray's, and Montrose, for which we offer our best thanks to the respective donors, at the same time regretting that the crowded state of our pages prevents our analysing them in the present Number.

